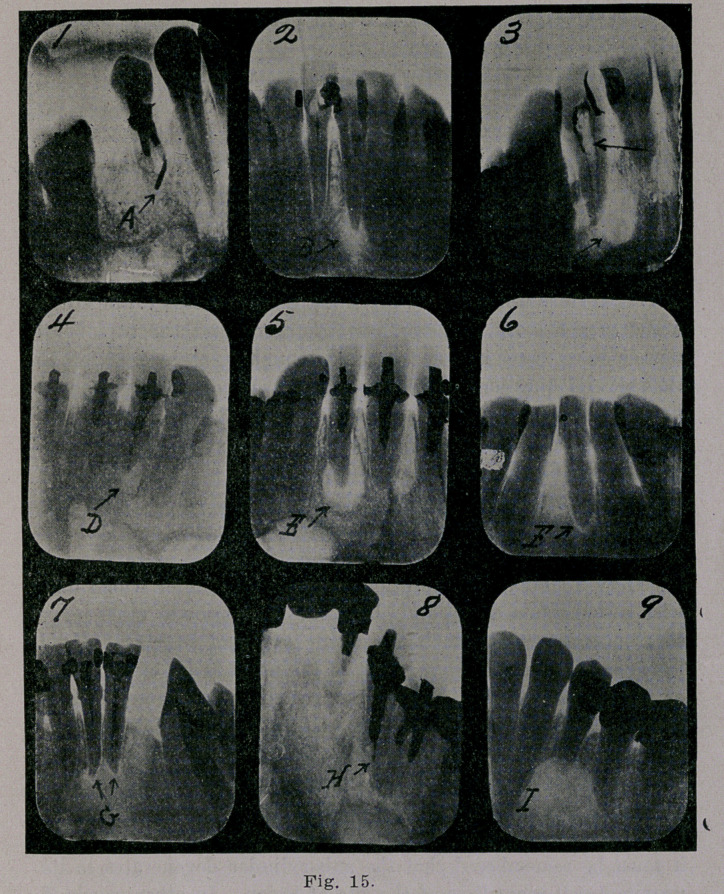# Indications for the Radiograph in the Study of Oral Conditions

**Published:** 1918-08

**Authors:** John M. Martin

**Affiliations:** Dallas, Texas


### ORIGINAL ARTICLES.

### Indications for the Radiograph in the Study of Oral Conditions.

BY J. M. MARTIN, M. D.

Professor of Electro-Therapeutics and X-Ray Methods, Medical Department of Baylor University and State Dental College; Radiographer to Baptist Memorial Sanitarium, Dallas, Texas.

The student of medicine has long been taught that most abnomal conditions about the mouth belong to the field of dentistry. The early dentist was a mechanic and gave little or no attention to pathological conditions, their cause, prevention or treatment. Billings, Rosenow and Thoma have brought to the medical and dental professions a wealth of knowledge regarding the subject of oral sepsis. Roentgenology has done its share in exposing the many and varied hidden recesses in which lurk the germs that produce the poisons that are responsible for many local and constitutional diseases. Alveolar and apicial absecesses were but a very short time ago treated with very little concern, and pyorrhea was not generally recognized until recently.

When I was handed my medical diploma in the spring of 1892 I had never heard of oral sepsis. I knew absolutely nothing concerning the infections that take place in the various sinuses of the head; neither did I know that the tonsils could be a source of infection in some distant part of the body. I had never heard that rheumatism, endocarditis and valvular lesions of the heart were frequently the result of some distant source of infection. The older physicians gave many medicines and used various kinds of liniments in their blind efforts to relieve or cure rheumatic pains. Yes, and many are prescribing the same remedies yet. Go into any drug store and look over the prescription files of today. You will find tongaline, salicylate of soda and many of the older remedies that were supposed to possess anti-rheumatic properties, in daily use. When an organ in the human body becomes the seat of pain, there is a cause. If the knee becomes inflamed, swolen and painful and the condition is diagnosed rheumatism, it is an error of judgment to pour medicines into the sufferer without first learning the cause and location of the infection. There may be many foci of infection and the finding and removal of one source may not give the patient any great amount of relief. When all of the sources of infection have been located and removed the rheumatic symptoms will clear up in a very short time. The greater part of the disturbance about the mouth has its origin in decayed teeth and is, in the main, the result of neglect on the part of the individual. Pyorrhea alveolaris (Rigg's Disease) is thought by some to be caused by the endomeba bucalis. This disease attacks the gums and extends down around the roots of the teeth through the peridental membrane. The teeth may or may not be decayed at first. Later, if the disease reaches the roots, the root canals and the pulp chambers become infected and the tooth dies. One or more apicial or alveolar absecesses discharging pus into the mouth and number of teeth denuded of their soft tissues and a portion of their alveolar support as a result of pyorrhea is a combination that cannot fail to undermine the health of the strongest individual. According to Rosenow, certain strains of bacteria, namely sterpcococus viridans, sterptococcus hemalyticus, staphylococus aures and albus are incubated in the mouth in vast numbers. These bacteria and their products are important factors in the causation of infection in various parts of the body. Teeming millions of these disease producing organisms are swallowed every hour in the day and night. These pathogenic bacteria find their way through the intestines into the lymph nodes of the mesentery from which there may be an active extension of the infection to the various abdominal organs. Through the blood stream the liver may become infected with an extension of the inflammation to the gall-bladder and the common duct. Inflammation in the lymph nodes may result in adhesions about the intestines. Appendicitis, ulcer and cancer of the stomach may be an indirect result of such infection. Though unable at this time to believe all that Rosenow teaches regarding the transition of one strain of bacteria through a cycle of various forms, producting distinctly different diseases, then coming back to the original strain; I am convinced that many of the acute and chronic infections of the circulatory, pulmonary and gastrointestinal tract, had their origin in and about the month, or accessory sinuses of the head. A filthy, foul-smelling mouth is a diseased mouth, and the respiratory and gastrointestinal tracts of such cases are sure to be infected to some considerable degree. The study of the teeth in health and disease is a field that the physician has neglected too long.. In a very imperfect way the author will try to bring to your attention a few of the most important conditions that may be found in the majority of mouths.

Fig. 1.

Fig. 1, is a schematic drawing of a normal tooth in longitudinal section. The enamel is intact, the gum fits up closely around the neck of the tooth, the peridental membrane is normal, the apex, the root canal and the pulp chamber are free from defects of any nature. Such a tooth serves the purpose for which it was intended and causes the possessor no discomfort. As a result of neglect the average mouth has several decayed teeth. The process of destruction is very slow at first. Though hard and dense as a flint the enamel, after long exposure to a deposit of food products, is caused to disintegrate. Once the enamel is broken or destroyed the less dense dentine becomes an easy prey to the inroads of the corroding deposit. The tooth now becomes sensitive to heat and cold and causes more or less discomfort at times. When the products of decay has reached the pulp chamber the real trouble begins. The infection travels on down the root canal to the apex where a granduloma or an abscess is formed.

Fig. 2.

Fig. 2, illustrates the three steps in the process of decay. The first and second steps may require months and even years but the third is usually of short duration, while the symptoms are more or less acute. While the cavity is small and well filled with products of food, the pain is usually slight. When the cavity is open and air and irritating liquids are allowed to reach the exposed nerve endings in the pulp chamber the pain is excruciating.

According to Thoma there are several varieties of alveolar and apicial abscess. There are likewise several steps in the formation of each variety.

Fig. 3.

Fig. 3, illustrates the early changes at the root following caries of the crown and infection of the pulp chamber and the root canal. The pus accumulated at the root is not allowed to escape through the root canal, pulp chamber and decayed cavity of the crown because this route is generally closed by particles of food, etc. The only course left is the destruction of the soft tissues which takes place along the course of least resistance.

In Fig. 4, the course of the abscess is toward and through the external alveolar process which gives away as the abscess grows larger.

Fig. 4.

In Fig. 5, the exterior of the alveolar process is reached and the periosteum is pushed away from the bone allowing the pus to accumulate, forming the ordinary gum boil. If the abscess is not lanced but is allowed to continue, the pus will burry its way outward through the gum as is illustrated in Fig. 6. Finally it opens into the mouth with its own accord but not until considerable tiseue destruction has taken place. This variety of apicial and alveolar abcess with the formation of a gum boil is the simpliest form. Though it may become chronic and continue to pour into the mouth a large amount of pus with thousands of bacteria. So long as the sinus remains open, there is very little discomfort and the victim may be entirely ignorant of the existence of such a condition in his mouth. One or all of the roots of a molar may be involved in an abscess that finds its way through the gum on the outer surface as is illustrated in Fig. 7. When the inner root alone is affected the the sinus is usually on the lingual side, as shown in Fig. 8. As is often the case the roots are just beneath the floor of the antrum. The pressure drives the pus in the line of least resistance and the antrum is entered and filled as pictured in Fig. 9. Though not so common as other forms of alveolar abscess it is more difficult to diagnose and is often overlooked by the attending physician and sometimes by the specialist. As a companion to the abscess that opens into the antrum, we sometimes see an alveolar abscess at the root of an incisor draining into the nasal cavity as illustrated in Fig. 10. This variety is exceedingly rare; having occurred but one time in the experience of the writer. An abscess at the root of a superior incisor may reach the surface through the alveolar pro-

Fig. 5.

Fig. 6.

Fig. 7.

cess and skin beneath the nose as seen in Fig. 11 The most unexpected sometimes happens and the pus may burrow its way downward through the inferior maxilla and find an opening below the chin as demonstrated in Fig. 12. An abscess at the root of a cuspid or bicuspid usually points on the labial side through the gum (Fig. 13) though it is not uncommon to find that the pus has worked its way downward between the periosium and the bone, opening through the skin on the lower border of the jaw about midway between the angle and the smyphisis, as illustrated in Fig. 14. This last variety the writer has seen a number of times. Extraction of the abscessed root usually cures the abscess and allows the sinus to heal, but not always. I have seen several fatal cases of carcinoma that had their origin in an alveolar abscess in the region of the cuspids. The history and clinical findings in most of these cases are conclusive, yet there are a large number of cases in which the diagnosis is in doubt.

Fig. 8.

Fig. 9.

Fig. 10.

The examination and interpretation by an experienced radiographer will furnish the dental surgeon with information that enable him to treat the case in the most scientific and practical way.

Fig. 11.

While the greater number of alveolar abscesses are due to caries of the teeth and diseases of the dental pulp, there are other causes that produce like symptoms, and because of the widely different etiology the treatment is different, therefore, a differential diagnosis is of the greatest importance.

Fig. 12.

Traumatic injury of the gums from any cause that is followed by infection involving the periostum or peridental membrane, may result in alveolar abscess. Injuries of this nature may result from an infected tooth brush, poor fillings, bands and crowns that do not fit but gouge and dig into the gums or otherwise irritate the gums by harboring large quantities of debris that can not be removed by patient or dentist by the ordinary methods of cleaning the teeth teeth. Such abscesses are usually shallow but the alveolar processes are just as surely destroyed and the support of the teeth is being little by little eaten away. The following group of radiographs from the author's collection will help to more forceably dmeonstrate the frequency and actual appearance of alveolar abscess as seen by the radiographer in the course of his routine work. In the place of alveolar abscess being the rare exception it is now almost the rule for we are able to find this condition, in some degree,

Fig. 13.

Fig. 14.

in almost every mouth.

The first radiograph in Fig. 15, shows a ralrge abscess that surrounds the root of the tooth leaving it with very little support. In filling the root canal it appears that the dentist bored through the side of the root as demonstrated by the forked appearance. A piece of the filling material has been forced out into the tissues as pointed out by arrow A. Whether this root should have been treated or the tooth pulled is a question that I will not attempt to answer. Had the dentist been supplied with a carefully made radiograph before this work was done I am sure that the treatment would have been different and I might add that the final results would have been more pleasing to the patient and more gratifying to the dentist. The second radiograph shows an abscess at the root of a lower central incisor with a beginning process around the root of its fellow. Both teeth are decayed and have been treated yet the root canals have not been filled. The process between the teeth is breaking down. In radiograph No. 3, the abscess is larger and the bone destruction is considerably greater.

The arrow indicates the root canal and the pulp chamber which is greatly enlarged and empty, the pulp having been destroyed. An abscess of this kind is capable of causing an enormous amount of infection which may be manifest in various parts of the body. An arthritis resulting from such a source could never be cured by means of local or constitutional treatment. The location of the source of infection suggests the treatment, i. e., the complete removal of the cause. Skiagraph No. 4, is of particular interest because the abscess indicated by arrow D is small and the tooth is filled to the tip of its root. The infection was probably well under way when the tooth was treated. In No. 5, we have the same condition as in the preceeding case, except that the destructive process is more extensive (arrow E) and the root canal is not filld. In No. 6, a tooth has been extracted and the crowns of the teeth are leaning toward each other with a considerable space between their roots where the alveolar process has been completely destroyed

Fig. 15.

and the tooth indicated by the arrow F is surrounded by the abscess and the tooth is without bony support. In No .7, pyorrhea has destroyed the alveolar process while an abscess surrounds the apices of both teeth as pointed out by the arrows at G. In No. 8, a large abscess lies beneath a splendid looking bridge, one end of which is tied on to a tooth that is completely undermined and rendered useless. In one of the laterals is seen a piece of broach (arrow H) extending beyond the end of the root. In No. 9, at 1, the abscess is rather large and involves the roots of three teeth and absorbing a part of the root of the central tooth. In each of the nine plates pyorrhea alveolaris is a complicating factor, if in fact, it is not the primary cause.

## Figures and Tables

**Fig. 1. f1:**
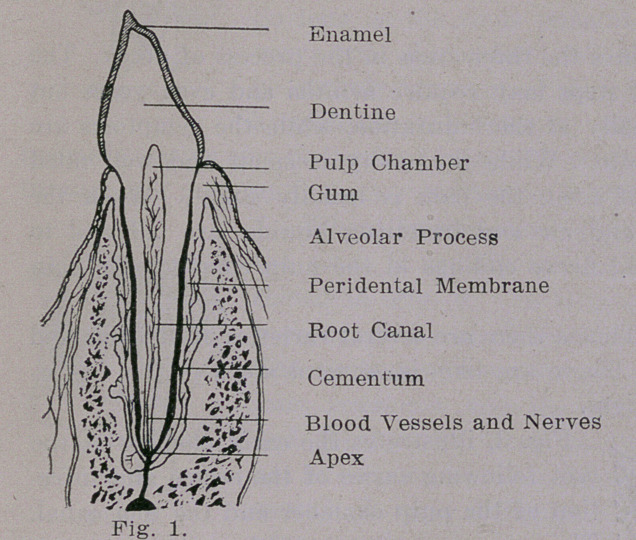


**Fig. 2. f2:**
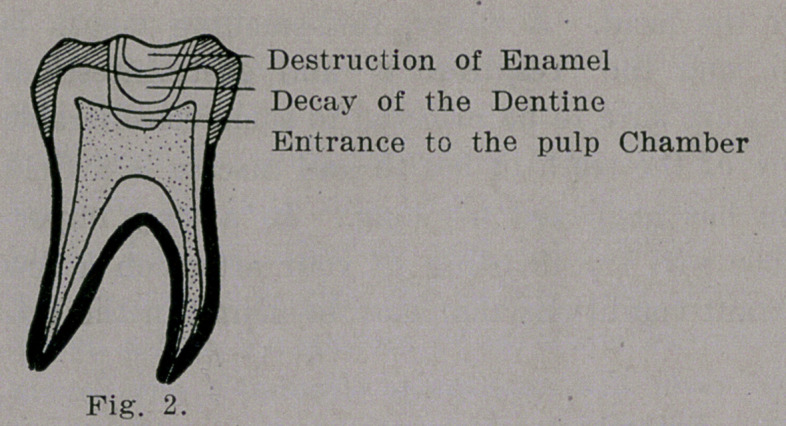


**Fig. 3. f3:**
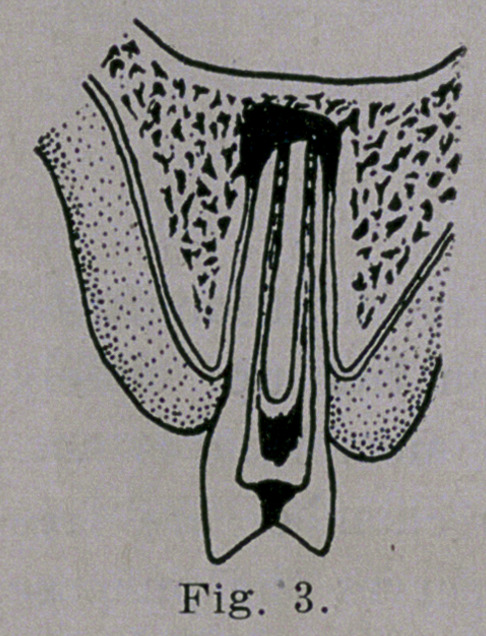


**Fig. 4. f4:**
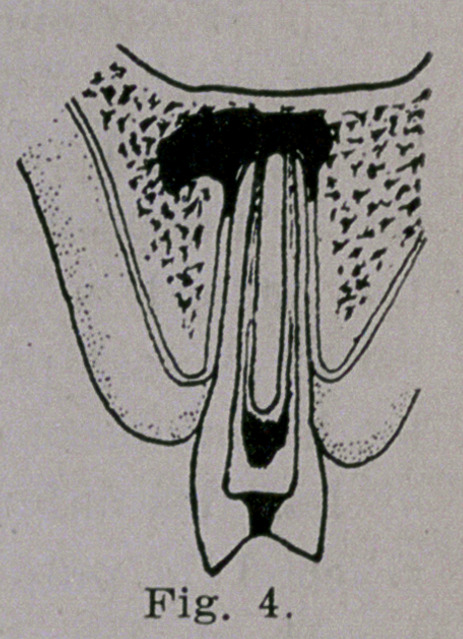


**Fig. 5. f5:**
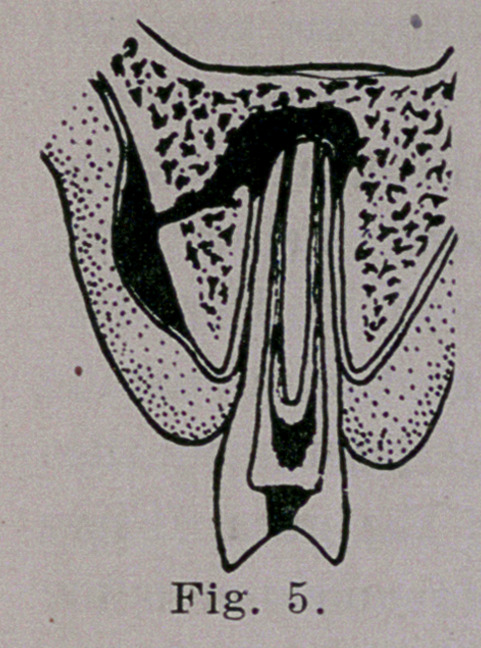


**Fig. 6. f6:**
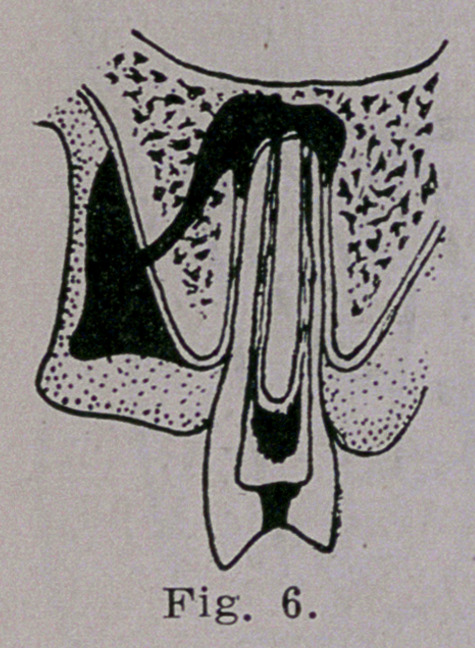


**Fig. 7. f7:**
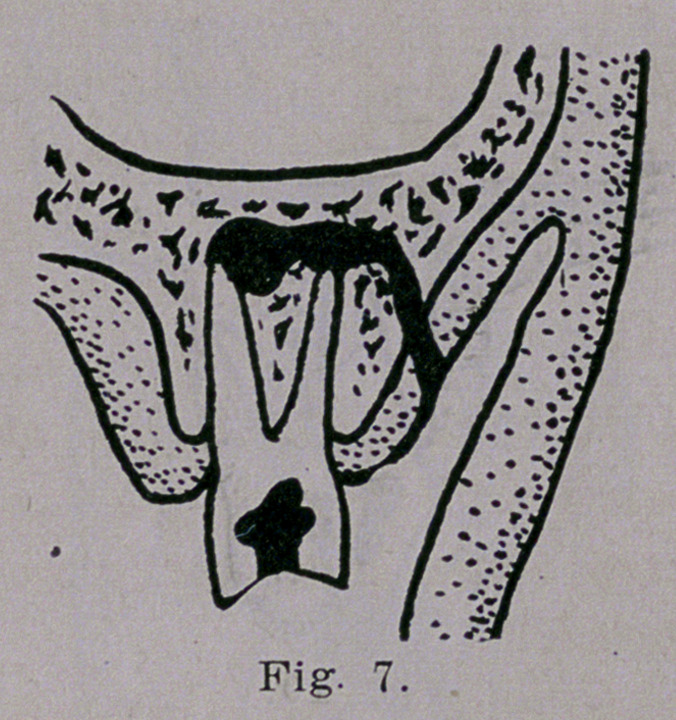


**Fig. 8. f8:**
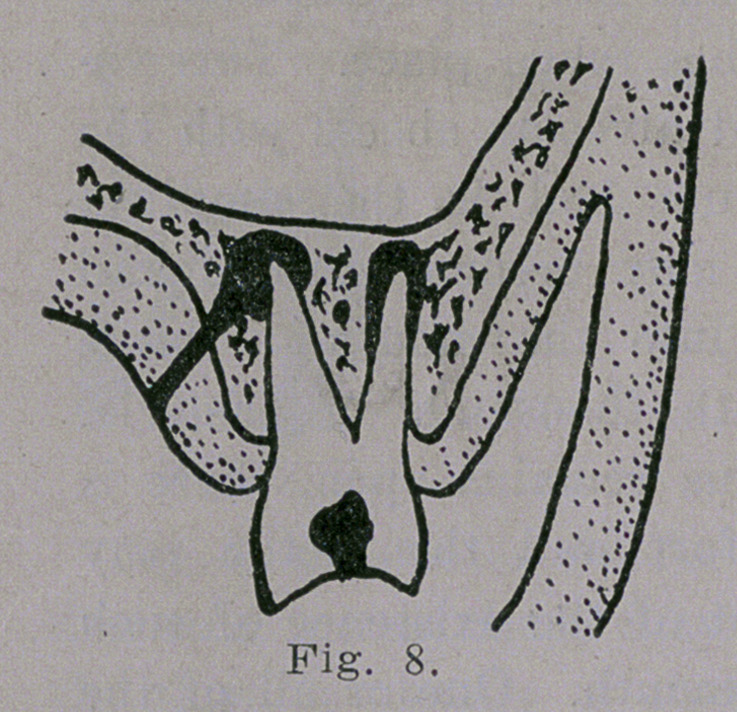


**Fig. 9. f9:**
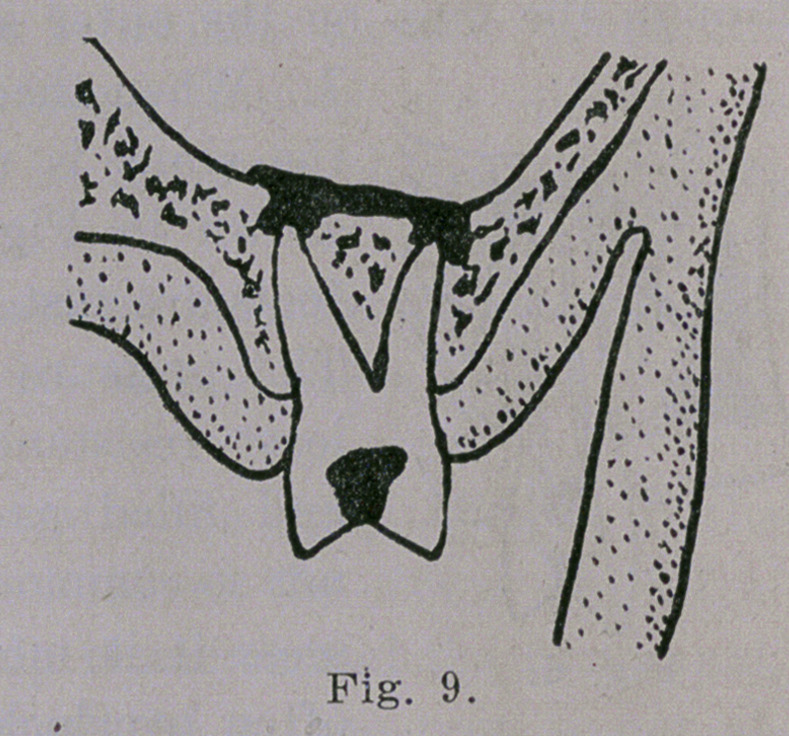


**Fig. 10. f10:**
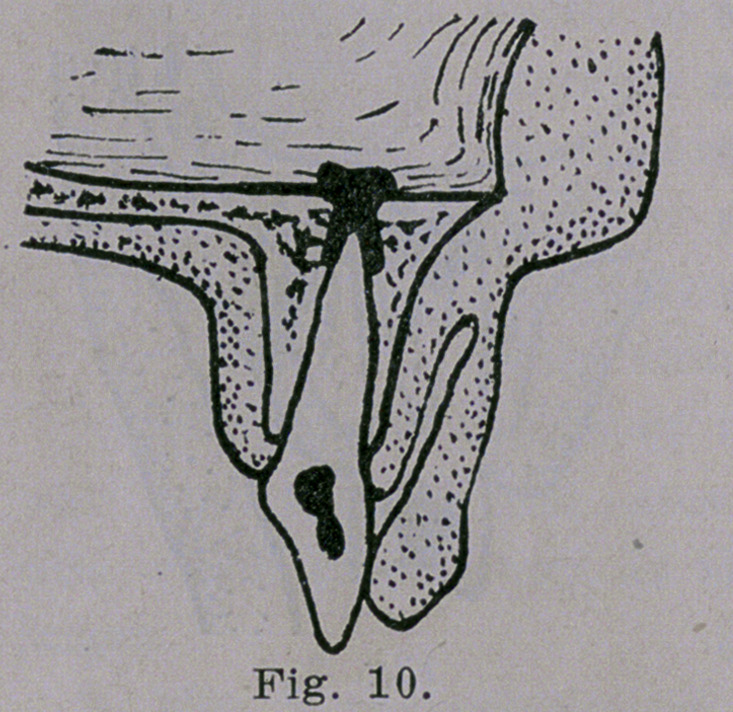


**Fig. 11. f11:**
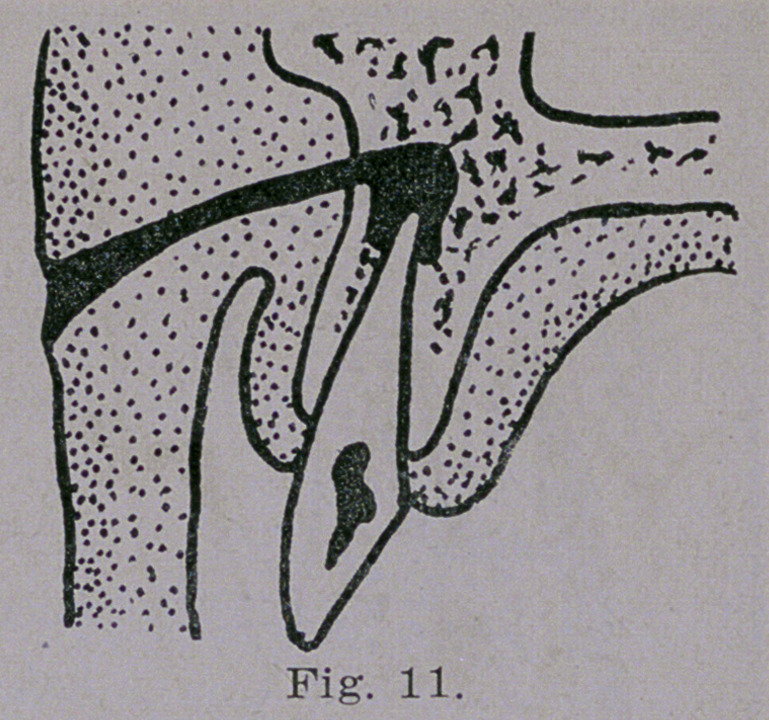


**Fig. 12. f12:**
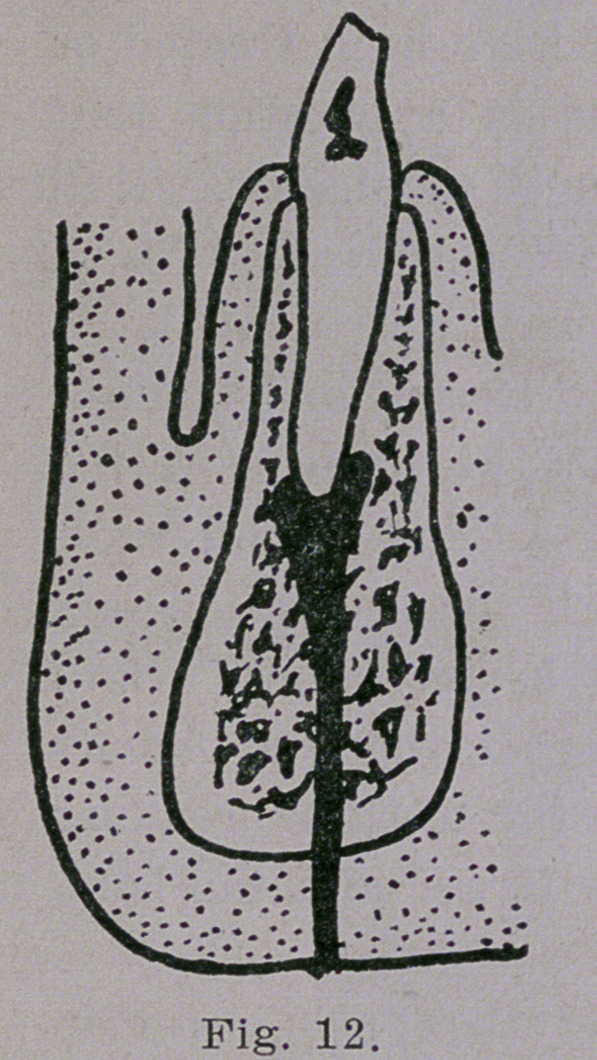


**Fig. 13. f13:**
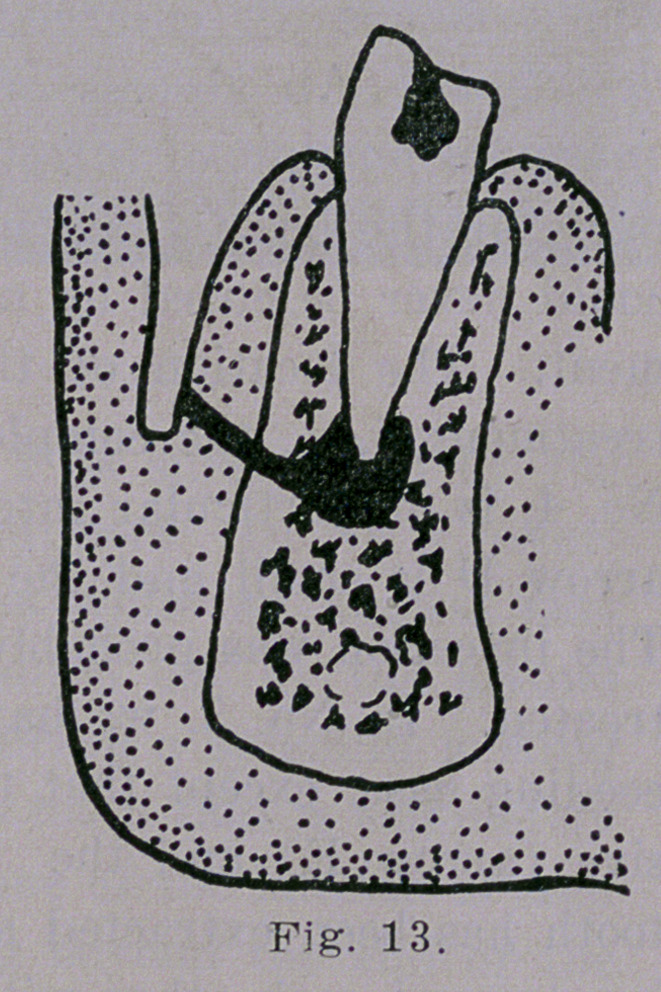


**Fig. 14. f14:**
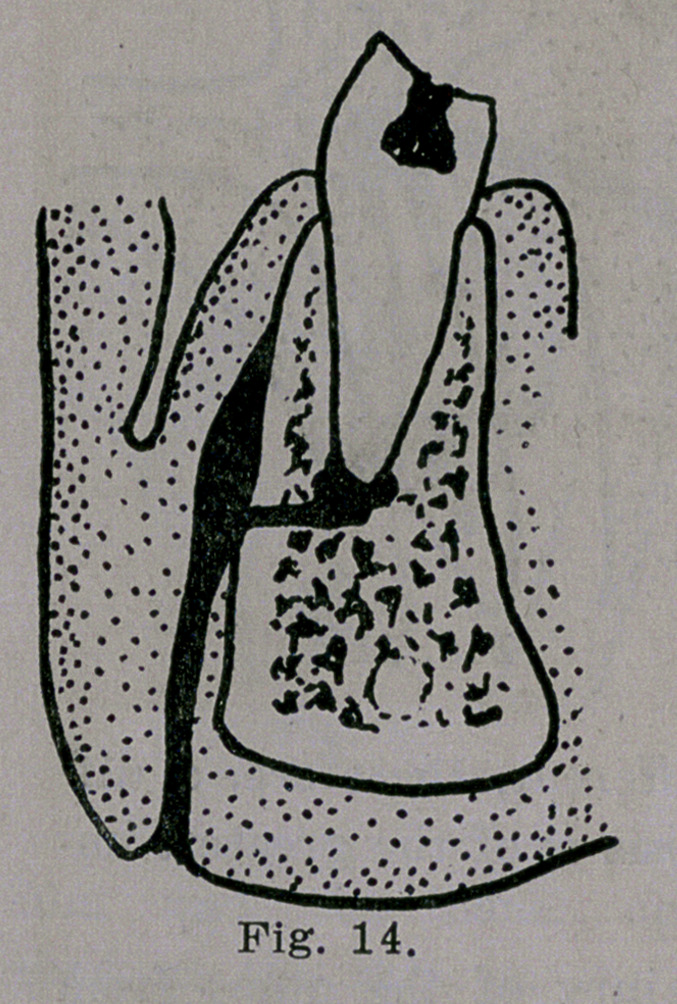


**Fig. 15. f15:**